# TargetMine, an Integrated Data Warehouse for Candidate Gene Prioritisation and Target Discovery

**DOI:** 10.1371/journal.pone.0017844

**Published:** 2011-03-08

**Authors:** Yi-An Chen, Lokesh P. Tripathi, Kenji Mizuguchi

**Affiliations:** 1 National Institute of Biomedical Innovation, Saito-Asagi, Ibaraki, Osaka, Japan; 2 Graduated School of Frontier Biosciences, Osaka University, Yamadaoka, Suita, Osaka, Japan; University of South Florida College of Medicine, United States of America

## Abstract

Prioritising candidate genes for further experimental characterisation is a non-trivial challenge in drug discovery and biomedical research in general. An integrated approach that combines results from multiple data types is best suited for optimal target selection. We developed TargetMine, a data warehouse for efficient target prioritisation. TargetMine utilises the InterMine framework, with new data models such as protein-DNA interactions integrated in a novel way. It enables complicated searches that are difficult to perform with existing tools and it also offers integration of custom annotations and in-house experimental data. We proposed an objective protocol for target prioritisation using TargetMine and set up a benchmarking procedure to evaluate its performance. The results show that the protocol can identify known disease-associated genes with high precision and coverage. A demonstration version of TargetMine is available at http://targetmine.nibio.go.jp/.

## Introduction

Advances in biomolecular research, coupled with rapidly increasing availability of information from multiple genome sequencing initiatives, global gene expression patterns, large scale molecular interaction experiments and genome wide association studies, have led to an exponential increase in biological data. The explosion of data, accompanied by a plethora of theoretical tools for predicting gene function, has created an information overload. The immense challenges in separating the biological wheat from the chaff have necessitated the development of a variety of analytical tools and databases to store and manage biological data and retrieve meaningful information to facilitate further experimental characterisation.

The biological role of a gene or a protein is not only defined by its sequence and structure but also by when and where it is expressed and its interactions with other biomolecules (such as proteins, nucleic acids and metabolites). In the post-genomic era, attempts at function annotation increasingly employ data from different types of repositories. Biological data from a single type of data source, though useful, is often limited in extent to which it may help uncover functional associations; either because of a systematic bias towards specific genes, gene families and pathways and/or inclusion of erroneous entries during data acquisition. With focus shifting from genes and proteins to biological systems, integrating information from multiple data types is a more robust and accurate means of enhancing existing interpretations and unravelling new functional associations as demonstrated in several studies [1], [2].

However, biological data integration is a formidable task. Different computational tools and data sources may often employ different approaches and formats for input, storing and retrieving relevant information that may often result in appreciable differences in data quality. This heterogeneity often restricts compatibility between different resources and limits the extent and efficiency of combined analysis. Furthermore, investigation of diverse data types necessitates a flexible, uniform and simplified interface to query, retrieve and analyse data across diverse sources. Despite these hurdles, the immense potential benefits of a combined investigative approach have spawned several initiatives towards integrated data repositories [3], [4], [5], [6], [7]. Among these, of particular interest are data warehouses, which compile all the relevant information to a common platform [6], [8], [9], [10], [11], [12], [13], [14]. A data warehouse is particularly desirable, since it permits a wide range of queries based on diverse attributes (including genes, proteins, families, pathways, ontologies, diseases and expression profiles) and possesses the ability to produce unified output and the flexibility in selecting the type and the order of the data sources. InterMine is a multi-purpose data warehouse framework (http://www.intermine.org/), originally developed for FlyMine, an integrated database for *Drosophila* and *Anopheles* genomics [13]. It features a sequence ontology-based data model and a user-friendly web interface permitting the end users to either design flexible and complex database queries, or choose from a library of ‘templates’ consisting of predefined queries with a simple form and description [13]. In addition, InterMine provides default parsers for integrating data from several resources with the framework for incorporating customised parsers and data sources. The flexibility in designing queries and integrating diverse data types provides a powerful tool for the researchers. In addition to FlyMine, InterMine also powers modEncode (http://intermine.modencode.org/), RatMine (http://ratmine.mcw.edu/ratmine/begin.do), YeastMine (http://yeastmine.yeastgenome.org:8080/yeastmine/begin.do) and MetabolicMine (http://www.metabolicmine.org/).

Identification of suitable targets (such as genes, proteins, non-peptide gene products and pathways) for characterisation is one of the most critical steps in biology, particularly in annotating gene function, drug discovery and understanding molecular bases of diseases. An integrated approach that combines results from multiple data types is best suited for optimal target discovery [15], [16]. The distinct merits of the InterMine framework have inspired us to develop TargetMine, an integrated resource for retrieval of target genes and proteins for experimental characterisation and drug discovery. In this paper, we describe the data sources available in the present release of TargetMine and their access and query capability. We also outline an objective protocol for target prioritisation with TargetMine that relies on the integration of diverse data types. Gene prioritisation refers to the selection of most interesting or promising genes from a larger set of genes for further analysis [17], [18]. Experimental evaluation of large gene lists to identify suitable candidates is a formidable and often impossible task and therefore, computational tools for candidate gene prioritisation have emerged over the years. These tools variously rely on functional associations, protein-protein interactions, gene expression data, sequence and structure properties or combinations thereof to select candidate genes [16], [17], [18], [19], [20], [21], [22], [23], [24], [25]. TargetMine was designed specifically for target prioritisation within the framework of a data warehouse and our prioritisation protocol, though less sophisticated than some standalone tools, is easier to use and provides flexibility in the choice of data sources that may be employed for analysis of query gene sets. Finally, we discuss the possibilities of future implementations in the TargetMine data warehouse to provide maximum coverage of the biological target space.

## Results and Discussion

### Data sources and Data models

A detailed description of the InterMine system is available elsewhere [13]. Here we restrict ourselves to a brief overview of the InterMine data organisation. InterMine is an open source data warehouse framework. Each entry in the system (such as a gene or a protein) is considered an ‘object’. The InterMine object-based data model, consists of ‘classes’ and reflects the relationships between different data types. Each class contains objects that share similar properties and a set of ‘attributes’ that correspond to various types of information (such as gene symbol and gene/protein identifier) associated with each object of that class. The classes are linked with each other by references that specify the associations between objects in different classes. The InterMine data structure readily allows the navigation of the stored biological data via the relationships between different data types, facilitated by an inbuilt tool termed ‘query builder’. The query builder tool permits the users to select and constrain the data types for the desired output. The list function enables the query process to be performed with a user-supplied list of objects and export the lists as either comma separated (csv) or tab separated values (tsv). It also permits the user to convert genes/proteins from one species to another based on KEGG orthology associations. The InterMine Web Service allows the users to query TargetMine from their own web pages and applications.

In addition to the existing InterMine classes, we have customised the InterMine data model and created new classes to collate biological data types most likely to help facilitate target discovery (Table S1). We will discuss some of these implementations below. As of now, the biological data in TargetMine for most part is limited to human, rat, mouse and fruit fly, the best studied model organisms in biology. The data sources compiled in TargetMine are summarised in Table 1.

**Table 1:** List of data sources in TargetMine.

Data	Organism*	Source
InterMine default		
Genome annotation	H, R, M, F	Entrez Gene
Protein annotation	H, R, M, F	UniProtKB
Protein domain	H, R, M	InterPro
Pathways	H, R, M, F	KEGG Pathway
Gene-gene interactions	H, R, M, F	BioGRID
GO annotation and the Gene Ontology	H, R, M	Gene Ontology, UniProtKB GOA
Data sources newly incorporated in TargetMine		
Protein 3D structure	Entire dataset	PDBe SIFTS
Protein-protein interactions	H	PPIview
Protein domain annotations	H, R, M	IPI
Structural classification	Entire dataset	SCOP
Orthologues / Paralogues	H, R, M, F, E	KEGG Orthology
Transcription factor	H	OregAnno, AMADEUS
Enzyme	H, R, M, F	The ENZYME database
Drug	H	DrugBank
Disease	H	OMIM†
Disease Ontology and DO annotation	H	Disease Ontology, BMC Genomics 10 Suppl 1:S6

*H: human, R: rat, M: mouse, F: fruit fly (*Drosophila*), E: *E. coli*.

† OMIM data are presently not distributed with the TargetMine demonstration version.

### Protein structures and domains

Structural data for biological macromolecules, especially proteins, have been extremely important in explaining their molecular and biochemical functions, evolutionary relationships and understanding their explicit biological roles [26]. It is well recognised that complementing protein sequence information with structural data is a robust approach towards more accurate protein function annotation [27] and hence, more reliable target discovery. However, integrating protein sequence and structural information from different sources remains a non-trivial task. In recognition of the obvious benefits of an integrated protein sequence-structure repository, we customised and embellished the default InterMine data model to combine protein sequence information from the UniProt database [28] with protein structure information from the Protein Data Bank (PDB) [29] and structural classification based on evolutionary relationships in the Structural Classification of Proteins (SCOP) database [30]. With our customised data model, the user can easily query for PDB structures cross-referenced (if available) with the protein of interest in the UniProt repository and other databases such as DrugBank [31] (e.g., “Show all the protein structures that contain the targets, as defined in DrugBank, of a given set of drugs” or “Given a list of proteins, show all the approved drugs solved in complex with any structure of these proteins if present”). The user can also retrieve disease associations, pathway associations and potential protein-drug associations, based on ligands associated with the protein structures, for the protein of interest (e.g., “Show all the PDB entries that contain a given drug”).

**Figure 1:**
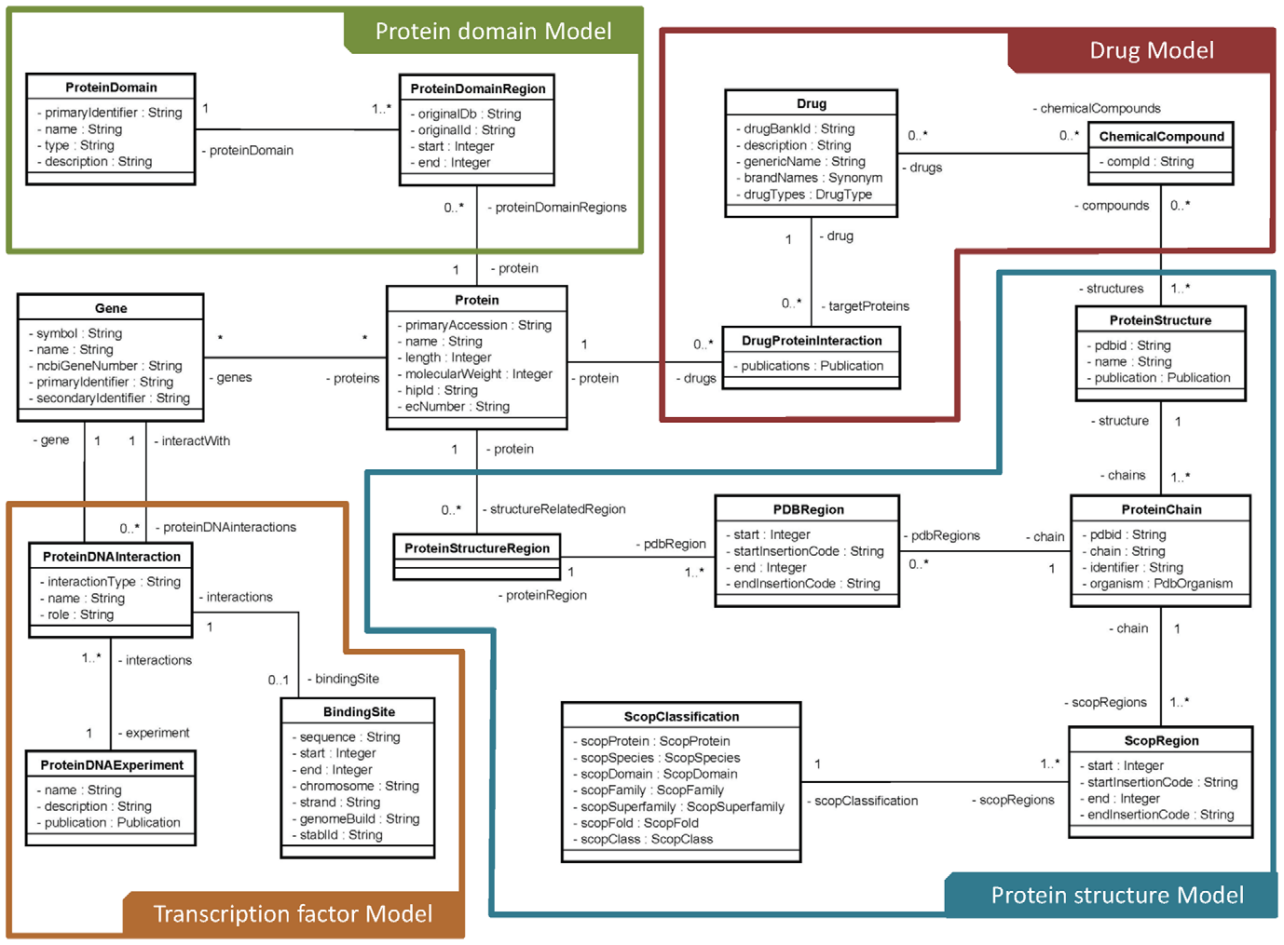
Schema for selected examples of newly created data sources in TargetMine. The data model is depicted as a class diagram in the Unified Modeling Language (http://www.uml.org). Some details of the model are ignored to reduce the complexity of the diagram.

Different data sources use different numbering systems for specifying protein regions. To associate protein sequences (in the Protein class) with protein structures (in the ProteinStructure class), we introduced two new classes (ProteinStructureRegion and PDBRegion; Figure 1). We also introduced the ProteinDomainRegion class to link the Protein class to the Protein domain class that stores InterPro [32] domain annotations. The PDB-UniProt mapping was taken from SIFTS [33] and InterPro domain assignments from IPI [34]. The integration facilitated querying detailed domain and structural assignments; for example, the user can query regions of a protein, for which structural information is available, and then retrieve domain annotations falling within these regions.

### Transcription factors

Transcription factors (TFs) are proteins that bind to specific DNA sequences, thereby regulating the expression (transcription) of their target genes [35]. TFs are of immense significance in biomedical investigations and some TFs such as nuclear receptors are important drug targets [36], [37]. In view of the significance of these protein-DNA interactions to cellular physiology, we modified the existing InterMine Interaction class, which describes gene-gene interactions, to define a new class named ProteinDNAInteraction. The ProteinDNAInteraction class contains specific attributes that reflect the unique aspects of protein-DNA interactions, such as protein (TF) binding sites in the regulatory regions of the target genes. These data were retrieved from AMADEUS [38] and OregAnno [39] resources and from assorted literature sources. Since different resources adopt different approaches to compiling protein-DNA interaction information, the combined source data were manually processed to uniformly assign Entrez gene identifiers to each participating gene and remove redundancies prior to the incorporation into TargetMine. The integration enabled us to make a complicated query such as: “Given a list of genes, retrieve all the TF-target relations observed within the list”.

### Other data classes

For disease and phenotype association, we created new classes and data parsers to retrieve the data from OMIM database [40] and human genome disease annotations [41]. Enzymes play key roles in many biological processes and are attractive candidates for experimental investigation aimed at understanding cellular processes, diseases and identifying suitable drug targets. We designed a new Enzyme class (linked to the Protein class) to gather all information on enzymes as curated in the Enzyme database [42]. The Enzyme class was also directly linked to the Pathway class by parsing the KEGG [43] mapping files, thereby providing links to their potential roles in cellular processes. Most genes and proteins function in association with other proteins and thus, the study of protein-protein interactions (PPIs) is critical to understanding their roles in living systems. In addition to the default InterMine Interaction class that was employed for storing biomolecular interactions from the BioGRID database [44], we designed a new ProteinInteraction class to collate all interactions curated in PPIview, an integrated repository of human PPIs [45]. This integration facilitated the querying of interacting partners of a gene/protein or a list of genes/proteins of interest and infer overall interaction networks involving these genes/proteins.

In addition, to expand the information space for sparsely annotated genes and proteins, we provided a framework for including *in silico* annotations derived from selected protein prediction tools (FUGUE [46], Protein-DNA binding propensity [47] and Protein-protein interaction sites [48]) and for including experimental data from in-house research.

### Target prioritisation and benchmarking

**Figure 2:**
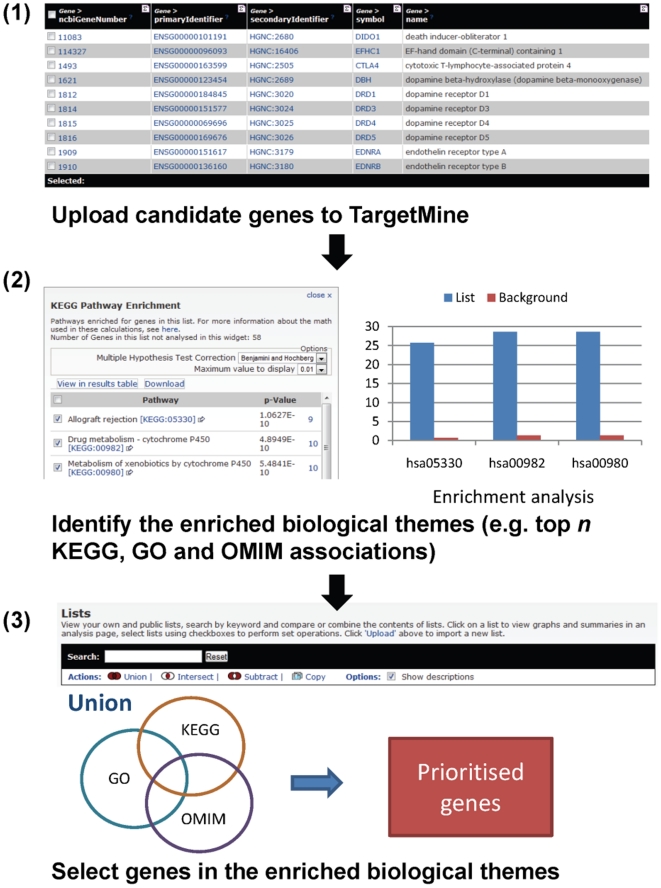
A schematic representation of the suggested objective protocol for candidate gene prioritisation with TargetMine.

Our general protocol for target prioritisation using TargetMine is shown in Figure 2. First, we upload a list of initial candidate genes or proteins (e.g., a set of differentially expressed genes or a set of proteins that interact with a given protein) to TargetMine to create a TargetMine gene list. Enrichment of specific biological themes (including but not limited to, KEGG pathways, Gene Ontology (GO) terms [49] and OMIM phenotypes) associated with the initial list is estimated by hypergeometric distribution and the inferred *p*-values are further adjusted for multiple test corrections to control the false discovery rate using the Benajmini and Hochberg procedure [50]. The significantly enriched biological associations (that satisfied, in this instance, a condition of *p*≤0.05 after a multiple test correction with the Benajmini and Hochberg procedure) can be visualised in the individual enrichment widgets. We gather the genes mapped to the top *N* significant associations (where *N* = 1,2,3…, an adjustable value reflecting incrementally relaxed thresholds) retrieved from KEGG (A), GO Biological Process (B) and OMIM (C) databases into separate lists and merge them (for example, by taking the union A
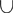
B
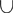
C of the retrieved genes) to infer corresponding sets of prioritised genes, albeit no ranking is provided at the moment. (We assume that an initial candidate list is from a single species and the enrichment calculation is performed using the data for this species only.)

**Figure 3:**
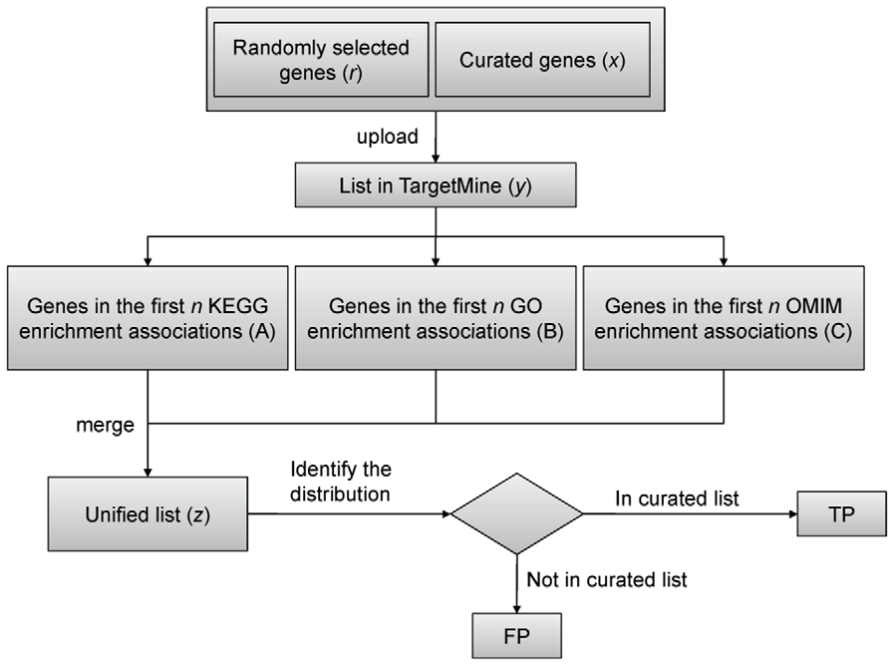
Outline of the procedure for benchmarking candidate gene prioritisation on 19 sets of known disease-associated genes with TargetMine. TP- True positive, FP- False positive (see text for details).

**Figure 4:**
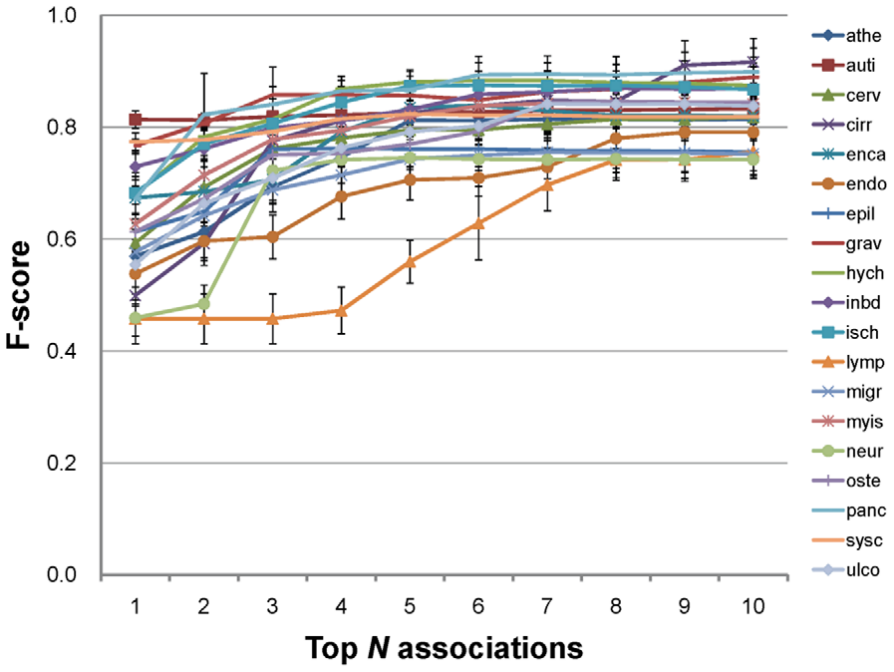
Benchmarking results for 19 sets of known disease-associated genes. (The full disease names and their abbreviations are listed in Table 2.) Each line represents the F-score for a particular disease data set as a function of the threshold (the top *N* significant associations considered). The error bars show the standard deviation across ten benchmarking evaluations for each disease.

To evaluate the effectiveness of TargetMine in identifying suitable targets for further characterisation, we performed target gene prioritisation tests (as described above) on 19 sets of known disease-associated genes compiled from the literature [51] (Table 2 and Figures 3 and 4; see Materials and Methods for details). In all instances, our prioritisation approach was supported by high sensitivity and precision values, and enforcing a threshold of collecting only the genes mapped to top seven associations (that satisfied a *p*-value cutoff of *p*≤0.05 after a multiple test correction with the Benajmini and Hochberg procedure) was by and large most suited to ensuring maximum coverage and minimum over-prediction (Table S2). Though for cirrhosis and cervical carcinoma, the number of false positives was slightly larger than those for the other diseases, the sensitivity and precision remained high.

**Table 2:** Benchmarking results for 19 sets of known disease-associated genes using top seven significant associations as the threshold.

*Disease*	*Sensitivity*	*Precision*	*F-score*
Atherosclerosis (athe)	0.786±0.04	0.848±0.06	0.814±0.03
Autism (auti)	0.824±0.02	0.839±0.07	0.830±0.03
Cervical carcinoma (cerv)	0.779±0.03	0.834±0.03	0.805±0.03
Cirrhosis (cirr)	0.850±0.02	0.848±0.05	0.848±0.02
Endometrial carcinoma (enca)	0.770±0.02	0.903±0.06	0.829±0.02
Endometriosis (endo)	0.621±0.07	0.897±0.07	0.729±0.03
Epilepsy (epil)	0.744±0.02	0.777±0.08	0.759±0.03
Grave's disease (grav)	0.803±0.01	0.934±0.04	0.863±0.02
Hypercholesterolaemia (hycl)	0.875±0.00	0.893±0.03	0.884±0.02
Inflammatory bowel disease (inbd)	0.897±0.04	0.838±0.08	0.863±0.04
Ischaemic stroke (isch)	0.909±0.04	0.845±0.08	0.874±0.04
Lymphoma (lymp)	0.636±0.10	0.788±0.06	0.697±0.05
Migraine (migr)	0.712±0.03	0.812±0.10	0.755±0.03
Myocardial ischemia (myis)	0.803±0.02	0.889±0.06	0.842±0.03
Neural tube defects (neur)	0.682±0.03	0.817±0.07	0.742±0.03
Osteoarthritis (oste)	0.822±0.02	0.870±0.05	0.844±0.02
Pancreatitis (panc)	0.923±0.05	0.874±0.07	0.895±0.03
Systemic scleroderma (sysc)	0.826±0.03	0.818±0.06	0.821±0.03
Ulcerative colitis (ulco)	0.856±0.02	0.831±0.08	0.841±0.04

We have repeated the tests by changing the proportion of known curated genes in an input gene list (from one third to one tenth). Although both sensitivity and precision decreased slightly, reasonable performance was maintained with a cutoff of six (Table S3), suggesting that the method still works for situations where only one tenth of input genes are disease-associated. We have also evaluated the results from a method using only a single data source. By taking the union of the collected genes from KEGG, GO and OMIM, the performance in most cases increased by about 0.1 points (measured by the F-score; see Materials and Methods), demonstrating the usefulness of the integration.

These results showed that the integration of diverse biological properties in TargetMine was a successful approach towards the identification of candidate genes for further investigation. Besides, the operation in TargetMine is semi-automatically accomplished by a few mouse clicks instead of preparing specific data files and running external software. The TargetMine data model permits retrieval of stored data and its analysis in a single interface and thus aids in efficient prioritisation. The ease of accomplishing such analysis via a simple web interface further underscores the utility of TargetMine as an effective tool in investigation of genes and genomes. In our benchmark tests, we chose KEGG, GO Biological Process and OMIM as the best sources for highlighting the functional associations of groups of genes but TargetMine also provides enrichment widgets for GO Molecular Function and Cellular Component, Drug and Disease Ontology (DO) associations, which may be used to assist in selecting candidate genes. The user may also employ TF-target associations to identify common regulatory themes that may be associated with a set of co-expressed functionally similar genes.

### Comparisons with other databases

As a data warehouse, TargetMine is not an alternative to large public databases (such as UniProt [28]) but rather, it is designed for use in individual laboratories in academia and industry. In comparison to existing integrated databases, TargetMine provides an alternative usage that aims to rapidly and efficiently retrieve varied biological information for large gene sets in a simplified manner. Most integrated databases are able to retrieve different biological properties, but are largely designed for simple queries for a single gene. Though some may provide facilities for batch query, the users in many instances need to employ external scripts for querying and post-processing the relevant data. In contrast, TargetMine provides a simple interface for batch query with numerous templates and the facility to construct complicated queries. The output options permit user-defined displays on the type and the order of different annotations. Besides, the enrichment widgets, as described above, provide a quick preliminary analysis of the genes in the list and thus, greatly help in understanding the enriched themes associated with query sets and also help complement the analysis performed by specialised gene prioritisation tools. Therefore, TargetMine facilitates biological data gathering and data analysis in a single user-friendly interface.

Although some commercial resources such as Ingenuity® (Redwood City, California) and MetaCore™ (GeneGo, St. Joseph, MI) provide more interaction and/or pathway data plus tools for statistical data analysis, they largely emphasise on collating gene annotations and mostly lack protein level annotations such as domains and structures. Additionally, several data types available in TargetMine such as Protein-DNA interactions, to the best of our knowledge, are not made available by other publicly available resources, some of which, including GeneDistiller [52] and PolySearch [53], can perform tasks similar to TargetMine's. However, the key difference is TargetMine's flexibility and its built-in prioritisation protocol; the data size and data types are readily customisable in TargetMine, providing a more flexible and comprehensive framework for target discovery.

TargetMine employs an “unsupervised” protocol for prioritisation, as opposed to most other comparable tools such as ToppGene [21] and Endeavour [20], which are “supervised” learning methods. Thus, while direct comparison with these other tools is difficult (and our data warehouse will complement, not replace, stand-alone tools), the preliminary results above suggest that TargetMine is well suited for target prioritisation. In our group, we have been using TargetMine for analysing a diverse array of experimental data and we have verified experimentally that some of the prioritised genes have been associated with the disease of interest [54].

### Future developments

TargetMine is structured to accommodate increasingly available biological data from large-scale experiments. Inclusion of new data sources would enable enhanced repertoire of functional associations currently available in TargetMine and at the same time expand the coverage to newer systems relevant to candidate gene prioritisation and drug discovery. We plan to add new data including host-pathogen interactions, specific gene and protein expression patterns, relationships between potential targets and chemical compounds and/or moieties, protein-compound interactions and single nucleotide polymorphisms (SNPs). We aim to supplement the newer data sources with further developments in the TargetMine web interface, lists, templates and tools for data visualisation (such as novel widgets) and analysis.

### Conclusion

TargetMine is an integrated data warehouse that enables complicated searches that are difficult to perform using existing comparable tools and therefore, assists in efficient target prioritisation. The benchmarking results for our proposed protocol for target gene prioritisation suggested the effectiveness of TargetMine in target discovery. The flexibility in TargetMine structure ensures that different types of biological data can be readily added and analysed to generate new hypotheses for further investigation. The inclusion of additional data sources and analytical tools will greatly enhance the ability of TargetMine to investigate biological systems for better target discovery.

## Materials and Methods

InterMine was downloaded from http://www.intermine.org. New parsers were written in Java and integrated into the InterMine code base. A list of URLs for the individual data sources can be found in Table S4. Part of OMIM data, not available in downloadable files, was retrieved from the online resource using custom PERL scripts and TF-target associations were manually processed prior to integration into TargetMine.

To benchmark our gene prioritisation protocol, we performed target gene prioritisation on 19 sets of known disease-associated genes (denoted by set *x*) compiled from the literature [51]. We first created test datasets (set *y*), where each curated gene set was merged with twice its number of unrelated randomly selected human genes (set *r*) to incorporate background “noise”. To avoid any bias incurred due to the selection of random genes, the process was repeated 10 times to infer 10 test gene sets for each curated gene list. The prioritisation tests (Figures 2 and 3) were then performed for each test gene set. We gathered the genes mapped to up to the top 10 associations, retrieved from KEGG, GO and OMIM databases to infer prioritised genes (set *z*). These were then compared with the curated gene sets (*x*∩*z*) and the efficiency of the prioritisation procedure was estimated with sensitivity and precision measures (Table S2). The True Positives (*TP*) in *z* were defined as genes present in *x*, while those corresponding to *r* were defined as False Positives (*FP*). The False Negatives (*FN*) were those genes corresponding to *x* that were not included in *z* at the specified threshold, while the True Negatives (*TN*) were genes corresponding to *r* correctly left out from the list of prioritised genes at a given threshold. Sensitivity, measuring the proportion of the known disease-associated genes that were correctly prioritised, was defined as *TP*/(*TP*+*FN*) and precision, measuring the proportion of the prioritised genes that were known disease-associated genes, was defined as *TP*/(*TP*+*FP*). The performance of the prioritisation protocol was also assessed using the F-score defined as 2(precision×sensitivity)/(precision+sensitivity) [55], [56].

## Supporting Information

Table S1 A full list of newly defined classes in TargetMine.
(XLS)

Table S2 Detailed benchmarking results for candidate gene prioritisation with TargetMine using 19 sets of known disease-associated genes.
(XLS)

Table S3 Detailed benchmarking results for candidate gene prioritisation with TargetMine using 19 sets of known disease-associated genes with increased background noise.
(XLS)

Table S4 A list of URLs for the individual data sources in TargetMine.
(XLS)

## References

[1] Ge H, Walhout AJ, Vidal M (2003). Integrating ‘omic’ information: a bridge between
genomics and systems biology.. Trends Genet.

[2] Gerstein M, Lan N, Jansen R (2002). Proteomics. Integrating interactomes.. Science.

[3] Burgun A, Bodenreider O (2008). Accessing and integrating data and knowledge for biomedical
research.. Yearb Med Inform.

[4] Chen LS, Emmert-Streib F, Storey JD (2007). Harnessing naturally randomized transcription to infer regulatory
relationships among genes.. Genome Biol.

[5] Garcia Castro A, Chen YP, Ragan MA (2005). Information integration in molecular bioscience.. Appl Bioinformatics.

[6] Stein LD (2003). Integrating biological databases.. Nat Rev Genet.

[7] Wong L (2002). Technologies for integrating biological data.. Brief Bioinform.

[8] Birkland A, Yona G (2006). BIOZON: a system for unification, management and analysis of
heterogeneous biological data.. BMC Bioinformatics.

[9] Cornell M, Paton NW, Hedeler C, Kirby P, Delneri D (2003). GIMS: an integrated data storage and analysis environment for
genomic and functional data.. Yeast.

[10] Helfrich JP (2002). Raw data to knowledge warehouse in proteomic-based drug
discovery: a scientific data management issue.. Biotechniques.

[11] Kasprzyk A, Keefe D, Smedley D, London D, Spooner W (2004). EnsMart: a generic system for fast and flexible access to
biological data.. Genome Res.

[12] Lee TJ, Pouliot Y, Wagner V, Gupta P, Stringer-Calvert DW (2006). BioWarehouse: a bioinformatics database warehouse
toolkit.. BMC Bioinformatics.

[13] Lyne R, Smith R, Rutherford K, Wakeling M, Varley A (2007). FlyMine: an integrated database for Drosophila and Anopheles
genomics.. Genome Biol.

[14] Shah SP, Huang Y, Xu T, Yuen MM, Ling J (2005). Atlas - a data warehouse for integrative
bioinformatics.. BMC Bioinformatics.

[15] Chen X, Jorgenson E, Cheung ST (2009). New tools for functional genomic analysis.. Drug Discov Today.

[16] Yang Y, Adelstein SJ, Kassis AI (2009). Target discovery from data mining approaches.. Drug Discov Today.

[17] Nitsch D, Goncalves JP, Ojeda F, de Moor B, Moreau Y (2010). Candidate gene prioritization by network analysis of differential
expression using machine learning approaches.. BMC Bioinformatics.

[18] Tranchevent Lo-C, Capdevila FB, Nitsch D, De Moor B, De Causmaecker P (2010). A guide to web tools to prioritize candidate
genes.. Briefings in Bioinformatics.

[19] Adie EA, Adams RR, Evans KL, Porteous DJ, Pickard BS (2006). SUSPECTS: enabling fast and effective prioritization of
positional candidates.. Bioinformatics.

[20] Aerts S, Lambrechts D, Maity S, Van Loo P, Coessens B (2006). Gene prioritization through genomic data fusion.. Nat Biotechnol.

[21] Chen J, Bardes EE, Aronow BJ, Jegga AG (2009). ToppGene Suite for gene list enrichment analysis and candidate
gene prioritization.. Nucleic Acids Res.

[22] Hutz JE, Kraja AT, McLeod HL, Province MA (2008). CANDID: a flexible method for prioritizing candidate genes for
complex human traits.. Genet Epidemiol.

[23] Kohler S, Bauer S, Horn D, Robinson PN (2008). Walking the interactome for prioritization of candidate disease
genes.. Am J Hum Genet.

[24] Lage K, Karlberg EO, Storling ZM, Olason PI, Pedersen AG (2007). A human phenome-interactome network of protein complexes
implicated in genetic disorders.. Nat Biotechnol.

[25] Perez-Iratxeta C, Wjst M, Bork P, Andrade MA (2005). G2D: a tool for mining genes associated with
disease.. BMC Genet.

[26] Joachimiak A (2009). High-throughput crystallography for structural
genomics.. Curr Opin Struct Biol.

[27] Watson JD, Laskowski RA, Thornton JM (2005). Predicting protein function from sequence and structural
data.. Curr Opin Struct Biol.

[28] The UniProt Consortium (2010). The Universal Protein Resource (UniProt) in 2010.. Nucleic Acids Res.

[29] Berman HM, Westbrook J, Feng Z, Gilliland G, Bhat TN (2000). The Protein Data Bank.. Nucleic Acids Res.

[30] Murzin AG, Brenner SE, Hubbard T, Chothia C (1995). SCOP: a structural classification of proteins database for the
investigation of sequences and structures.. J Mol Biol.

[31] Wishart DS, Knox C, Guo AC, Cheng D, Shrivastava S (2008). DrugBank: a knowledgebase for drugs, drug actions and drug
targets.. Nucleic Acids Res.

[32] Hunter S, Apweiler R, Attwood TK, Bairoch A, Bateman A (2009). InterPro: the integrative protein signature
database.. Nucleic Acids Res.

[33] Velankar S, McNeil P, Mittard-Runte V, Suarez A, Barrell D (2005). E-MSD: an integrated data resource for
bioinformatics.. Nucleic Acids Res.

[34] Kersey PJ, Duarte J, Williams A, Karavidopoulou Y, Birney E (2004). The International Protein Index: an integrated database for
proteomics experiments.. Proteomics.

[35] Latchman DS (1997). Transcription factors: an overview.. Int J Biochem Cell Biol.

[36] Overington JP, Al-Lazikani B, Hopkins AL (2006). How many drug targets are there?. Nat Rev Drug Discov.

[37] Nebert DW (2002). Transcription factors and cancer: an overview.. Toxicology.

[38] Linhart C, Halperin Y, Shamir R (2008). Transcription factor and microRNA motif discovery: the Amadeus
platform and a compendium of metazoan target sets.. Genome Res.

[39] Griffith OL, Montgomery SB, Bernier B, Chu B, Kasaian K (2008). ORegAnno: an open-access community-driven resource for regulatory
annotation.. Nucleic Acids Res.

[40] McKusick-Nathans Institute of Genetic Medicine JHUB, MD) and National
Center for Biotechnology Information, National Library of Medicine
(Bethesda, MD) (2010).

[41] Osborne JD, Flatow J, Holko M, Lin SM, Kibbe WA (2009). Annotating the human genome with Disease
Ontology.. BMC Genomics.

[42] Bairoch A (2000). The ENZYME database in 2000.. Nucleic Acids Res.

[43] Aoki-Kinoshita KF, Kanehisa M (2007). Gene annotation and pathway mapping in KEGG.. Methods Mol Biol.

[44] Stark C, Breitkreutz BJ, Reguly T, Boucher L, Breitkreutz A (2006). BioGRID: a general repository for interaction
datasets.. Nucleic Acids Res.

[45] Yamasaki C, Murakami K, Fujii Y, Sato Y, Harada E (2008). The H-Invitational Database (H-InvDB), a comprehensive annotation
resource for human genes and transcripts.. Nucleic Acids Res.

[46] Shi J, Blundell TL, Mizuguchi K (2001). FUGUE: sequence-structure homology recognition using
environment-specific substitution tables and structure-dependent gap
penalties.. J Mol Biol.

[47] Ahmad S, Gromiha MM, Sarai A (2004). Analysis and prediction of DNA-binding proteins and their binding
residues based on composition, sequence and structural
information.. Bioinformatics.

[48] Murakami Y, Mizuguchi K (2010). Applying the Naive Bayes classifier with kernel density
estimation to the prediction of protein-protein interaction
sites.. Bioinformatics.

[49] Ashburner M, Ball CA, Blake JA, Botstein D, Butler H (2000). Gene ontology: tool for the unification of biology. The Gene
Ontology Consortium.. Nat Genet.

[50] Noble WS (2009). How does multiple testing correction work?. Nat Biotechnol.

[51] Chen J, Xu H, Aronow BJ, Jegga AG (2007). Improved human disease candidate gene prioritization using mouse
phenotype.. BMC Bioinformatics.

[52] Seelow D, Schwarz JM, Schuelke M (2008). GeneDistiller–distilling candidate genes from linkage
intervals.. PLoS One.

[53] Cheng D, Knox C, Young N, Stothard P, Damaraju S (2008). PolySearch: a web-based text mining system for extracting
relationships between human diseases, genes, mutations, drugs and
metabolites.. Nucleic Acids Res.

[54] Tripathi LP, Kataoka C, Taguwa S, Moriishi K, Mori Y (2010). Network based analysis of hepatitis C virus Core and NS4B protein
interactions.. Mol Biosyst.

[55] Hripcsak G, Rothschild AS (2005). Agreement, the f-measure, and reliability in information
retrieval.. J Am Med Inform Assoc.

[56] van Rijsbergen CJ (1979). Information retrieval.

